# The Role of Pericytes in Regulation of Innate and Adaptive Immunity

**DOI:** 10.3390/biomedicines11020600

**Published:** 2023-02-17

**Authors:** Siarhei A. Dabravolski, Elena R. Andreeva, Ilya I. Eremin, Alexander M. Markin, Irina I. Nadelyaeva, Alexander N. Orekhov, Alexandra A. Melnichenko

**Affiliations:** 1 Department of Biotechnology Engineering, Braude Academic College of Engineering, Karmiel 2161002, Israel; 2 Laboratory of Cell Physiology, Institute of Biomedical Problems, Russian Academy of Sciences, 123007 Moscow, Russia; 3 Petrovsky National Research Center of Surgery, 119991 Moscow, Russia; 4 Institute for Atherosclerosis Research, 121609 Moscow, Russia; 5 Institute of General Pathology and Pathophysiology, 125315 Moscow, Russia

**Keywords:** pericyte, immune system, immunosuppression, leukocyte trafficking

## Abstract

Pericytes are perivascular multipotent cells wrapping microvascular capillaries, where they support vasculature functioning, participate in tissue regeneration, and regulate blood flow. However, recent evidence suggests that in addition to traditionally credited structural function, pericytes also manifest immune properties. In this review, we summarise recent data regarding pericytes’ response to different pro-inflammatory stimuli and their involvement in innate immune responses through expression of pattern-recognition receptors. Moreover, pericytes express various adhesion molecules, thus regulating trafficking of immune cells across vessel walls. Additionally, the role of pericytes in modulation of adaptive immunity is discussed. Finally, recent reports have suggested that the interaction with cancer cells evokes immunosuppression function in pericytes, thus facilitating immune evasion and facilitating cancer proliferation and metastasis. However, such complex and multi-faceted cross-talks of pericytes with immune cells also suggest a number of potential pericyte-based therapeutic methods and techniques for cancer immunotherapy and treatment of autoimmune and auto-inflammatory disorders.

## 1. Introduction

Pericytes (PCs) are perivascular multipotent cells wrapping microvascular capillaries, where they contribute to vascular development, maturation, remodelling, architecture and permeability, collaborate the functional integrity of the blood–brain barrier (BBB), participate in tissue formation and regeneration, and regulate blood flow [1,2]. PCs originate from human pluripotent stem cells (HPSCs), they are heterogeneous in their morphology, distribution, embryonic origin, and identification markers. Many markers have been used to identify PCs, including nerveglial antigen-2/chondroitin sulfate proteoglycan 4 (NG2), α-smooth muscle actin (αSMA), platelet-derived growth factor receptor β (PDGFR-β), endoglin (CD105), aminopeptidase N (CD13), the regulator of G-protein signaling-5 (RGS5), desmin, the adhesion molecule CD146, and others [3]. However, not all PCs express every single marker, many of these markers are not exclusive to PCs and their expression is dynamic and varies between different organs, developmental stages, or pathological states [4]. Because of the common origin, PCs share some properties and markers with MSCs multipotent mesenchymal stem cells (MSCs) and smooth muscle cells (SMCs), PCs can trans-differentiate into other cells of the mesenchymal lineage (such as myocytes, osteocytes, chondrocytes, and adipocytes) and neural cells, which make proper PC identification and tracking a very challenging task [5]. The commonly used markers for PC identification include NG2, *α*SMA, PDGFR-*β*, RGS5, and desmin; however, the set of used markers would vary depending on the research task, used species, organ/tissues of origin, developmental stages and pathological conditions (Table 1) [6].

The role of pericytes in angiogenesis, vascular homeostasis, and the blood–brain barrier relies on intensive communication with surrounding cells, most importantly with endothelial cells (ECs) through several well-characterised signalling pathways, such as Jagged Canonical Notch Ligand 1/Notch Receptor 3 (Jag1/Notch3), Platelet Derived Growth Factor Subunit B (PDGF-B/PDGFR-β) and Angiopoietin 1/TEK Receptor Tyrosine Kinase (Ang-1/Tie-2) [13,14]. Moreover, pericyte-like cells establish a continuous three-dimensional network in subendothelial intima [15,16]. Cells in such a network actively communicate with each other through the gap junctions and create multicellular strictures in a form of clusters [17].

Pericytes also play a role in cancer biology, where they contribute to the processes of tumour angiogenesis and metastasis, which are crucial for cancer progression and metastasis [18]. A recent finding suggested that the immune system can promote resistance to anti-angiogenic drugs. Considering known association between tumour angiogenesis and immune cells, the number of therapies applying combined application of immunosuppressive and anti-angiogenic drugs were investigated (such as pazopanib–nivolumab, sunitinib–nivolumab, atezolizumab–bevacizumab) [19,20] and reviewed in [21,22].

Although PCs have been mainly studied by neuroscientists because of their crucial role in the BBB maintenance and spinal cord injury repair [23,24], the frontiers of pericyte research are constantly expanding, opening their new physiological and pathological function and attracting more attention from research groups worldwide. Beyond the field of angiogenesis research, the pericytes are involved in diabetes mellitus associated complications [25], atherosclerosis [26] and other cardiovascular diseases [27]. In this review, we focus on the recent progress in our understanding of the far less explored role of pericytes in immune system regulation. Moreover, we discuss how pericytes contribute to the tumour cells’ immune evasion mechanisms and thereby facilitate tumour development and progression.

## 2. Pericytes as Macrophage-like Cells

Early research suggested that PCs represent a macrophage-like non-professional APC antigen-presenting cell (APC), which actively participates in immune responses. The abilities for phagocytosis and pinocytosis were documented for PCs, along with the presence of markers: T Cell Surface Glycoprotein CD4 (CD4), alpha chain of the integrin Mac-1/CR3 (CD11b), leukocyte-common antigen (CD45), scavenger receptors Macrophage Antigen CD68 (CD68), Macrophage-Associated Antigen (CD163), Fc receptors, and major histocompatibility complex (MHC) class II molecules. However, results of this pioneering research later were questioned because of the absence of clear PC identification, so the results might be referred to perivascular macrophages (reviewed in [28]). In the case of standard histological sections used for light microscopy, it was not possible to discern the vascular wall from the perivascular space, thus the cells of the adjacent compartments (such as perivascular macrophages or juxtavascular microglia) were often misconceived as ‘‘pericytes’’. Later introduced practice required morphological and localisation features and the presence of at least two markers (such as NG2 and PDGFR-*β*) to distinguish PCs from other surrounding cells [29]. Indeed, Interferon Gamma (IFN-γ) and Tumour Necrosis Factor-Alpha (TNF-α) treatments induced MHC class II molecules and up-regulated CD68 expression, and increased phagocytosis in PCs [30]. However, in other research IFN-γ treatment induced the expression of MHC class II in cultured PCs, but not the co-stimulatory molecules CD80 or CD86 [31], which have a crucial role in modulating T cell immune function [32].

Recent research demonstrated that a subpopulation of brain pericytes originated from phagocytic macrophages during vascular development. Functionally matured macrophages, expressing CD31+ F4/80+ CD206 and CD11b markers were shown to trans-differentiate into NG2/PDGFRβ/desmin-expressing cerebrovascular pericytes, which cover the subventricular vascular plexus in the very early phase of central nervous system vascular development [33]. Furthermore, use of the in vivo fate-tracing technologies showed that myeloid lineage progenitor cells contribute to pericyte development in embryonic skin vasculature. TGF-β signalling initiates the differentiation process in culture, and Tgfbr2 mutants exhibit deficient pericyte development in skin vasculature [34].

Similarly, macrophage-like properties were recently described also in peripheral tissues. NG2+ cells were increased in ischaemia-reperfusion (I/R)-injured kidneys and expressed macrophage markers CD11b and F4/80 had phagocytic activity and expressed anti-inflammatory cytokines (mannose receptor and IL-10). Furthermore, intravenous transfusion of renal NG2+ cells isolated from donor mice reduces renal damage and facilitated renal recovery from I/R injury [35].

In summary, recent reports have confirmed the presence of macrophage-specific markers and some macrophage-like properties on PCs. However, these reports are dedicated to the PCs associated with specific developmental stage, tissue, and/or pathological conditions. Therefore, future research is required to define the exact molecular mechanism connecting PC and macrophage. This knowledge may provide new approaches for the treatment of several neurodevelopmental disorders and injuries of brain and peripheral organs in the future.

## 3. Pericytes in the Regulation of the Innate Immune System

### 3.1. PC in Inflammatory Responses

Inflammation is one of the first responses of the innate immune system to infection, cellular damage, or toxic compounds. These factors cause the release of pro-inflammatory stimuli (such as cytokines, chemokines, interferons, growth, and cytotoxic factors) which recruit immune cells to the site of inflammation and promote removal of pathogen, tissue healing, and damage regeneration [36].

PC actively responds to pro-inflammatory stimuli (mainly IFN-γ, IL-1β, and TNF-α) by secreting diverse cytokines and chemokines, such as IL-6, CXC (CXCL1, CXCL8, and CXCL10), and CC (CCL2, CCL3, and CCL5) [37,38]. Moreover, IL-17-activated PCs produce granulocyte colony-stimulating factor (G-CSF) and granulocyte-macrophage colony-stimulating factor (GM-CSF), thus prolonging neutrophil survival. Interestingly, IL-17-stimulated PC also enhanced neutrophils’ phagocytic capacity and induced neutrophil synthesis of IL-1α, IL-1β, TNF-α, MIF (macrophage migration–inhibitory factor), and CXCL8. Therefore, PC secretome can lure diverse professional immune cells (monocytes, macrophages, Th1, CD8, and NK cells) to the site of inflammation [38,39].

Interestingly, human brain PCs respond on the Transforming Growth Factor Beta 1 (TGFβ1) treatment by up-regulation of some inflammatory-related genes (*IL-6*, Cyclooxygenase-2 (*COX2*), Matrix Metallopeptidase 2 (*MMP2*) and NADPH Oxidase 4 (*NOX4*)) and down-regulation of others (*IL-8*, Monocyte Chemoattractant Protein-1 (*MCP-1*), Vascular Cell Adhesion Protein-1 (*VCAM1*), and *CXCL1*). TGFβ1 acts through Mad-Related Protein (SMAD2/3) transcription factors, which regulate many cellular processes, such as cell proliferation, apoptosis, and differentiation. Therefore, TGFβ1 treatment reduces PC proliferation and phagocytic ability, and expression of the scavenger receptors (CD36, CD47, and CD68) [40]. PC treatment with lipopolysaccharide (LPS) stimulates release of mostly pro-inflammatory cytokines and chemokines (IL-1a, IL-2, IL-6, IL-7, IL-8, GM-CSF, macrophage colony-stimulating factor (MCFF), CCL-5 (RANTES), CCL-17 (TARC), growth-regulated protein alpha/beta/gamma (GROa/b/g) and stromal cell-derived factor 1 alpha (SDF-1a). On the contrary, PC treatment with platelet-derived growth factor-BB (PDGF-BB) leads to release of growth factors (brain-derived neurotrophic factor (BDNF), basic fibroblast growth factor (b) (FGFb), nerve growth factor beta (βNGF), vascular endothelial growth factor (VEGF) and placental growth factor (PLGF)), and IL-12 (p40/70), IL-1,3 and IL-15, and decreases secretion of tumour necrosis factor alpha (TNFα). These data confirm that PC secretome greatly depends on the exogenous stimulus and plays an important role in the regulation of the inflammation process [41].

Recent research demonstrated functional connection between TGFβ1 and PDGF-BB signalling pathways. As it was shown, a mice model of focal cerebral ischemia with postnatally induced systemic PDGFR-knockout had BBB dysfunction (represented with deformed TJ (tight junction) and decreased expression of TJ proteins, ample endothelial transcytosis, severe brain edema and neurologic functional deficits) and reduced TGFβ1 expression. PDGF-BB treatment increases the SMAD2/3 expression, while TGFβ1 antibody partially inhibits Smad2/3 phosphorylation. Furthermore, TGFβ1 treatment mitigates TJs reduction, neurologic dysfunction, and edema formation in mutant mice’s model of focal cerebral ischemia [42]. These data support the importance of PDGF-BB/TGFβ signalling axis for pericyte network functionality and BBB integrity in cerebral ischemia and suggest a potential therapeutic target for ischemic stroke (reviewed in [43]).

The presence of a specific marker might help to define specific function and/or phenotype of PC. As it was shown on CD73+ CD45− human brain PC, two populations can be distinguished by high/low CD90 expression. CD90+ PC demonstrated higher proliferation rates and sensitivity to the TGFβ1 action in comparison to CD90− PC. On the other hand, CD90− PC showed a contractile and high extracellular matrix (ECM)-producing phenotype, accompanied with greater pro-inflammatory response to LPS and IFN-γ stimulation than CD90+ cells [44].

Despite active involvement in the inflammatory responses, PCs also have an anti-inflammatory C/EBPδ-based mechanism. CCAAT enhancer binding protein (C/EBP) transcription factors participate in many physiological processes and known as context-dependant regulators of inflammation [45,46]. Treatment of brain NG2+, SMA+, PDGFRβ+, and C/EBPδ knockdown PC with IL-1β enhanced production of Intercellular Adhesion Molecule 1 (ICAM), IL-8, MCP-1, and IL-1β, and reduced expression of Superoxide Dismutase 2 (SOD2) and COX2. These results suggest that PCs have a C/EPB-based mechanism to limit peripheral immune cells infiltration and prevent further inflammatory responses [47].

PCs are involved in immunological responses under inflammatory conditions through secretion of inflammation-related proteins, which include context-dependent release of both pro-inflammatory and anti-inflammatory factors. However, further research is required to better understand the molecular mechanism connecting exogenous stimuli and PC secretome in the regulation of inflammatory responses.

### 3.2. PC in Innate Immunity

The innate immune system is the first non-specific line of defence against pathogens. Several classes of receptors known as pattern-recognition receptors (PRRs) are responsible for sensing pathogens, damaged host cells, and associated products. There are two PRR classes: pathogen associated molecular patterns (PAMPs) which are associated with pathogen recognition, and damage-associated molecular patterns (DAMPs), which are associated with components of damaged, apoptotic, and senescent host cells [48]. PRRs are mainly expressed by the EC (endothelial cells), dendritic cells, monocytes, macrophages, neutrophils, and other specialised cells. Among known PRR families toll-like receptors (TLRs) and NOD-like receptors (NLRs) are the most studied [49]. Further in this section we review a recent publication describing PRRs expression by PCs and suggesting their active role in innate immune responses.

The expression of TLR4 in human brain PCs was detected at a low level in unstimulated human brain PCs, and LPS treatment increased its expression. On the contrary, the treatment with High mobility group box 1 (HMGB1), a pro-inflammatory mediator and a comparatively weak TLR4 activator, had no such effect. However, both LPS and HMGB1 effectively stimulated production of diverse chemokines, cytokines, and adhesion molecules: IL-6, IL-8, CXCL1, CXCL2, CXCL3, CCL2, SELE, ICAM1, and VCAM1 by LPS in an NF-κB-dependent way, and IL-8, CXCL1, CXCL2, CXCL3, and CCL2 by HMGB1. Subsequently, increased expression of adhesion molecules by LPS treated PC resulted in an increased adhesion of peripheral blood leukocytes, thus confirming the role of PC in the inflammatory cascade [37]. Recently, activation of TLR4-NfκB-mediated pro-inflammatory cascade in brain PCs by free long-chain fatty acids (LCFAs) was shown. Interestingly, LCFAs-mediated activation was specific for PC where it resulted in a breakdown of neural microvasculature and neuroinflammation [50].

Further research demonstrated that human brain PCs also express the peptidoglycan (PGN)-sensing Nucleotide Binding Oligomerization Domain Containing 1 (NOD1) receptor. Interestingly, while both receptors have been shown to effectively elicit a pro-inflammatory response upon stimulation, NOD1 and TLR4 receptors acted through the separated signalling pathways. The NOD1 signalling cascade involved Receptor Interacting Serine/Threonine Kinase 2 (RIPK2), which was not identified in the TLR4 pathway [51]. Finally, in vitro experiments with human brain PCs demonstrated expression of multiple PRRs (such as NOD1 and 2, NLRC5, NLRP1-3, NLRP5, NLRP9-10, and NLRX, TLR2, TLR4-6, and TLR10) in control conditions. PC stimulation with IL-1β and TNF-α up-regulated the expression of *IL-1β*, *TLR2* and *TLR9-10*, *NLRC4* and 5, *NLRP5* and 10, and *NOD2* genes. On the other hand, oxidative stress up-regulated only *TLR10* and *NLRP9* genes [52]. Similarly, the important role in innate immune responses was described also for lung PCs in vitro and in vivo. As it was shown, lung PCs expressed several functional TLRs (TLR1-2, TLR4, and TLR6-7), which facilitate IL-6, CXCL1, CCL2, and ICAM-1 induction upon LPS stimulation [53].

PCs also contribute to the activation of the complement system, a crucial component of the innate immune response. As it was shown on the mice model of kidney fibrosis, PCs subjected to obstructive or folic acid injury secrete Complement C1q Chain A (C1q), pro-inflammatory cytokines (IL-6, Fibroblast Secretory Protein (FSP), MCP-1, and Macrophage Inflammatory Protein-1-Alpha (MIP-1α)), extracellular matrix components, collagens (α1, α2, (I and XII), fibrillin-1, proteoglycans such as fibronectin, cathepsins B and D, fibulin-2, osteopontin, thrombospondins 1 and 2), and increased Wnt3a-mediated activation of Wnt/β-catenin signalling, which are hallmarks of myofibroblast activation [54]. Recently, the role of PCs in producing Complement Component 5a Receptor 1 (C5aR1) in renal fibrosis was shown. PC-specific deletion of C5aR1 in Foxd1Cre+/− mice reduced folic acid-mediated kidney fibrosis. Moreover, the secretion of several cytokines (such as IL-6 and MIP-2) was reduced in comparison with control PCs [55]. In total, these data suggest that the secretion of complement components (C1q and C5a/C5aR1) by PCs contributes to the development of renal fibrosis, thus the inhibition of complement activation represents a potential therapeutic target in treatment of kidney fibrosis and chronic kidney disease.

For a long time, PCs have been considered as passive players, responsible for the sensing of the pro-inflammatory factors released by specialised immune cells and amplification of inflammatory responses. However, the expression of functional PRRs and the ability to secrete cytokines, chemokines, and adhesion molecules suggest that PCs are important contributors to the innate immune responses. Therefore, modulation of the PC pro-inflammatory activities may provide a novel strategy to reduce vascular injury in a variety of pathological conditions.

### 3.3. PC in the Regulation of Immune Cell Trafficking

Leukocyte trafficking to the inflammation sites is orchestrated by adhesion molecules and chemokines, which guide immune cells through ECs, the basement membrane, and PC sheath to enter the perivascular space [56]. Further, we discuss recent works which have demonstrated how PCs regulate the recruitment of leukocyte to inflammation sites.

**Figure 1:**
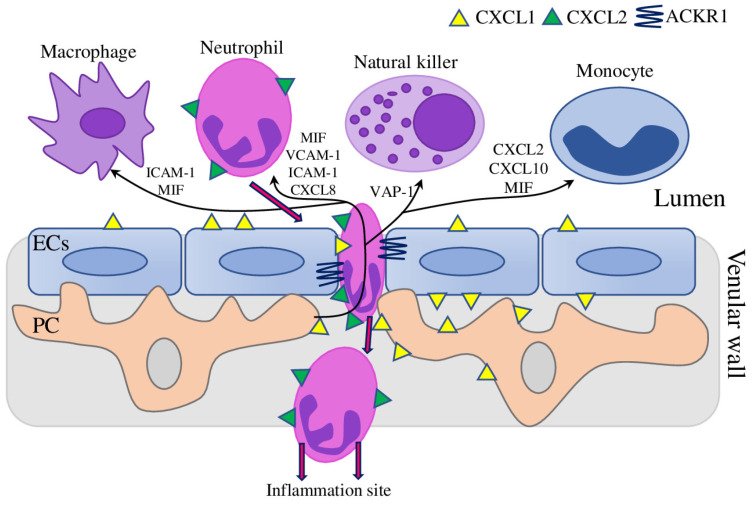
The role of pericytes in leukocyte transmigration across the endothelial barrier. Stimulated pericytes induce the expression and release of various factors, attracting and facilitating trafficking of leukocytes across vessel walls (black arrows). In particular, neutrophils navigate through the venular walls with CXCL1 and CXCL2 chemokines, produced by ECs/PC and neutrophils, respectively, and EC junctions-enriched expression of CXCL2 receptor ACKR1 (atypical chemokine receptor 1) (magenta arrows).

In the cellular model of pyogenic meningitis, perivascular macrophages sense pathogens and generate an inflammatory cascade, which was amplified by human brain vascular PCs and translocated across the endothelial barrier to act on circulating neutrophils (Figure 1). Bacteria were unable to stimulate PCs and ECs directly, while macrophages-originated cytokines up-regulated transcription of multiple neutrophil chemokines in PC and significantly enhanced neutrophil transmigration across the endothelial barrier. At the same time, the permeability of the endothelial barrier to small molecules was not changed, which suggests that the mechanism of PC-mediated chemokine translocation is highly specific and can be the most effective therapeutic target to reduce neutrophil-mediated pathology in pyogenic meningitis [57].

Similarly, the crucial role of PCs in the control of leukocyte trafficking into the CNS central nervous system (CNS) during autoimmune neuroinflammation was demonstrated in the adult pericyte-deficient mice (Pdgfb^ret/ret^) model of EAE (experimental auto-immune encephalomyelitis). PC-deficient mice die from the massive influx of immune cells into the brain upon EAE induction. However, the severity of atypical EAE symptoms of Pdgfb^ret/ret^ mice was reduced by treatment with anti-VCAM-1 and anti–ICAM-1 antibodies, suggesting that absence of PC promoted neuroinflammation. Additionally, the massive influx of leukocytes into the brain was accompanied by the increased level of myelin peptide-specific peripheral T cells in the circulation, which leads to the development of spontaneous neurological symptoms [58]. These results are in accordance with another study, which demonstrated decreased survival and increased ratios of early apoptosis in PC incubated with serum of mice with EAE [59]. These data suggest that PC dysfunction within brain vasculature can drive the development of a neuroinflammatory disorders.

PC-neutrophil interactions play a crucial role in mediating pathologic neutrophil recruitment and microvascular remodelling in neutrophilic dermatosis, an inflammatory disease characterised in vivo by increased levels of TNFα, IL-17A, and collagen IV, and decreased the level of laminin. As it was recently shown, mast cells are the major source of IL-17A, thus providing a novel mast cells-IL-17A-PC axis [60]. In the presence of TNFα and IL-17A, in vitro culture of human placental PCs demonstrated enhanced collagen IV and fibronectin production, thus actively remodelling the basement membrane to facilitate neutrophil recruitment. Furthermore, interactions between PCs and leukocytes in vitro up-regulate the expression of MMPs, in particular MMP-3, leading to the degradation of vascular fibronectin and laminin, thus confirming results observed in vivo and the causative role of PCs in this process [61]. On the molecular level, the neutrophils navigate through the venular walls with CXCL1 and CXCL2 chemokines. Upon TNFα stimulation, CXCL1 was mainly produced by ECs and PC, thus promoting luminal and sub-ECs neutrophil crawling. CXCL2 was mainly produced by neutrophils and was crucial for correct breaching of EC junctions through the binding of atypical chemokine receptor 1 (ACKR1), in which expression was enriched within EC junctions. Thus, CXCL1 and CXCL2 facilitate efficient migration of neutrophils through venular walls into inflammatory sites [62].

Similarly, recent research demonstrated the role of Vascular Adhesion Protein-1 (VAP-1), an inflammation-inducible adhesion molecule, in interactions between endometrial PC and uterine natural killer (uNK) cells in vitro. Endometrium PCs were located around the spiral arterioles and constitutively expressed VAP-1, which was required to maintain their clonogenic, adhesive, migratory, and contractile properties. On the contrary, VAP-1 knockdown reduces the number of uNK cells stably adherent to the PC. These data provide a timely characterisation of endometrial PCs and suggest a vital role of VAP-1 in regulating the trafficking of innate immune cells in the human endometrium [63].

Interestingly, one of the most widely used PC markers was recently shown to modulate expression of ICAM-1, and subsequently regulate leukocyte adhesion and transmigration. NG2 silencing in the human placental PC increased ICAM-1 expression in ERK1/2-mediated way. Furthermore, the inverse expression pattern of NG2 and ICAM-1 was confirmed also in an in vivo mice model and two glioblastoma cell lines, suggesting that these results are not specific to a particular cell line or model system and can be considered as therapeutic targets to modulate ICAM-1-mediated immune responses [64].

Recently, the key role of the PC-monocyte cross talk in cocaine-mediated neuroinflammation was shown [65]. Treatment of human brain vascular PCs with cocaine resulted in up-regulation of the pro-inflammatory cytokines (IL-1β, IL-6, and TNFα) and secretion of CXCL10 chemokine, which increased monocyte transmigration in both in vitro and in vivo experiments. These results provide a novel role for PCs as a cocaine-responsible cells promoting monocyte recruitment in CXCL10-dependent way [65,66].

In total, these findings demonstrated that cross-talks of PCs with different types of leukocytes (macrophage, neutrophil, natural killer, and monocyte) modulate their trafficking through vessel walls with chemokines and adhesion molecules (CXCL1, CXCL2, CXCL10, VCAM-1, ICAM-1, MIF, and VAP-1) (Figure 1). Further investigations of factors regulating such cross-talks and development of interventions aimed at blocking or restrict the migration of specific leukocyte type through PC could be a promising therapeutic strategy to abrogate inflammation.

## 4. Pericytes in the Regulation of the Adaptive Immune System

The adaptive immune response is antigen-specific and generates pathogen-specific responses mediated through B and T types of lymphocytes. Thus, B cells are involved in the humoral immune response, while T cells in the cell-mediated immune response, where T-killer sub-type cells recognise antigens coupled to Class I MHC molecules and T cells of the helper and regulatory sub-types, recognise only antigens coupled to Class II MHC molecules (reviewed in [67]).

Based on the high expression levels of immune inhibitory receptor ligand PD-L1 Programmed Cell Death 1 Ligand 1 (PD-L1) and PD-L2, PCs have been proposed to act as immunosuppressors. Furthermore, the treatment of MHC class II+ human placental PC with IFN-γ cannot stimulate cell proliferation or cytokine production through the resting allogeneic CD4 T cells, instead it renders CD4 T cells clonally anergic [68]. These results were also confirmed on retinal PC, which inhibited activated T cell proliferation and inflammatory cytokine production [69].

The role of PC in immunosuppression is of critical significance in the transplantation area because the rejection of allogeneic organs by the host immune system is mediated by infiltration of circulating host T cells into the graft [70]. Indoleamine 2,3-dioxygenase 1 (IDO1) and forkhead box P3 (FOXP3) have been identified as major players in PC-mediated immunosuppression. IDO1 catalyses the first and rate-limiting step in tryptophan catabolism to N-formyl-kynurenine and plays a vital role in different pathophysiological processes such as immunoregulation, anti-tumour, and anti-microbial defence (reviewed in [71,72,73]). FOXP3 acts as a major regulator of the development and function of Tregs (regulatory T cells), which usually turns the immune response down [74]. Thus, an excess of Tregs activity helps cancer cells to avoid the immune system, while a deficiency of Tregs activity is important in autoimmune and autoinflammatory disorders, and other pathological processes and diseases [75].

As it was recently shown, unstimulated PCs can directly present alloantigen to TEM (effector memory T cells), while IFN-γ–activated PCs instead suppress TEM proliferation, but not cytokine production or signalling. Further investigation revealed that IFN-γ treatment induced in PC significantly higher up-regulation of IDO1 in comparison with IFN-γ treated ECs. The levels of IDO1 correlated with tryptophan depletion in vitro and IDO1 knockdown reduces immunosuppressive properties of IFN-γ treated PCs, thus suggesting that immunosuppressive properties of human PCs result from IFN-γ-induced IDO1-mediated tryptophan depletion [76].

Similarly, PCs of different origin (human brain, placenta, or derived from human pluripotent stem cells) incubated with nonactivated peripheral blood T cells mediated a significant increase in the frequency of allogeneic CD25highFoxP3+ regulatory T cells. Moreover, PC induced de novo formation of functional CD4+CD25highFoxP3+CD127−, suppressive regulatory T cells. Furthermore, PC-mediated induction of CD25highFoxP3+ Tregs over T cell activation is regulated by the secretion of TGF-β and constitutive expression of PD-L1. Implanted PC mixed with CD4+CD25− T cells into NOD/SCID immunodeficient mice maintained a non-immunogenic phenotype and mediated the development of functional regulatory T cells. Thus, these data allow to distinguish PC-mediated immunomodulation from immunosuppression and suggest application of PC in allogeneic cell therapy without provoking immediate immune responses and actively modulating suppressive immunity [31].

### PC in Allergic Asthma and Pulmonary Fibrosis

Allergic asthma is a chronic and potentially life-threatening pulmonary disease characterized by the constriction of airways, airway wall thickening, and overall reduction in airflow and increased airway hyperresponsiveness, driven by a Type 2 immune response to inhaled allergens. Despite steadily growing asthma prevalence over the past few decades, the advances in the pharmacology and disease management allow to improve the patients’ outcome and reduce the hospitalisation rate [77]. The large airway remodelling (excess mucus and ECM production, hyperplasia, hypertrophy, and hyper-sensitisation of airway smooth muscle) causes physiological symptoms (such as wheeze and cough) [78]. Research has established the central role of PCs in allergic asthma and pulmonary fibrosis tissue remodelling.

In pathophysiological conditions associated with allergic asthma, PCs have been shown to uncouple from the airway microvasculature and accumulate in the subepithelial region of chronically inflamed airways. These PCs demonstrated elevated expression of the myofibroblast marker α-smooth muscle actin (α-SMA) and contributed to airway hyperactivity in an allergen-driven model of chronic allergic asthma mice model [79]. Furthermore, lung PCs trans-differentiate into myofibroblasts in the presence of TGFβ signalling and causes fibroblastic foci. Interestingly, the such PC-originated myofibroblasts can evade apoptosis, which suggests that therapeutic action on pericyte–myofibroblast transition may be a more effective strategy than to kill activated myofibroblasts for the treatment of pulmonary fibrosis [80]. Another mechanism promoting PCs into myofibroblast transition was defined in the bleomycin lung injury mice model. After intratracheal bleomycin treatment, the expression of integrin, contractile, and secretory markers, alongside many diseases and tissue-remodelling gene sets in PC was increased. Additionally, the extracellular matrix ligand (Arginine, Glycine, and Aspartate (RGD)) in fibronectin was identified as the key potentiator of PC into myofibroblast transition [81]. Further research into the molecular mechanisms regulating PC behaviour during pulmonary fibrosis may provide new promising diagnostic and therapeutic tools for lung fibrosis and other tissue remodelling diseases.

**Figure 2:**
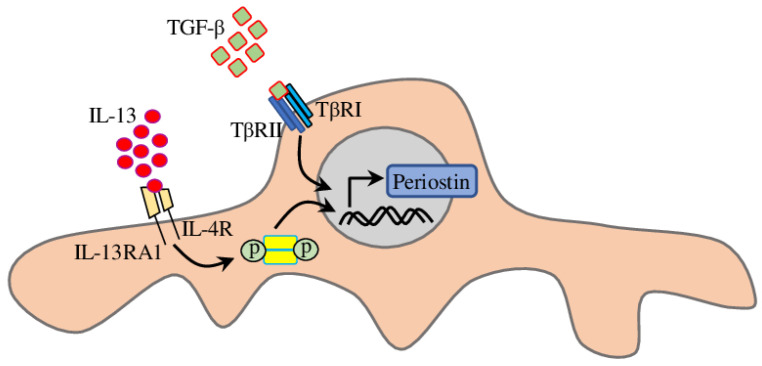
Schematic representation of the proposed signalling pathways regulating periostin expression in PC.

Periostin, a matricellular protein isolated from osteoblasts and strongly expressed in the periosteum [82], was recently recognised as an inflammatory mediator associated with TGFβ and implicated in PC migration and development of a variety of different allergic diseases [83], including asthma [84]. Recent research demonstrated that PCs express periostin with increased production after TGFβ or IL-13 treatment or exposure of mice to house dust mite. Moreover, PC treated with periostin were more migratory, which is the key event in airway wall remodelling in allergic airway disease (Figure 2) [85].

Chemoattractant receptor-homologous molecule expressed on TH2 cells (CRTH2) is a G-protein-coupled receptor expressed on human T helper type 2 (Th2) cells, eosinophils, mast cells, and basophils, its major ligand is the mast cell product prostaglandin D2 (PGD2). PGD2 binding to CRTH2 initiates allergic inflammation and eosinophil activation. Moreover, single nucleotide polymorphism of the *CRTH2* genes is associated with asthma development, susceptibility, and increased Th2 cell differentiation [86]. Because of the crucial role of the CRTH2 in activation of allergic inflammation, CRTH2 agonists are widely recognised as a promising therapeutic target, especially in case of Th2 inflammation-driven asthma [87]. However, CRTH2 agonists have demonstrated a rather limited efficiency if clinical trials [88,89]. Interestingly, recent preclinical research has found that *CRTH2* is expressed in PC, which are protected in CNS by a blood–brain barrier and not exposed to drugs from circulation. Therefore, application of specific therapeutic anti-CRTH2 antibody led to depletion of *CRTH2*-expressing circulating basophils and eosinophils, while CRTH2 expression on PC was not affected and did not cause vascular damage. These data suggest a new role of PCs in allergic inflammatory diseases and a wider set of methods for therapeutic interventions [90].

In total, PCs are important players in regulation of the adaptive immunity. IDO1 and FOXP3 are the major regulators of PC-mediated immunosuppression and immunomodulation. Further investigation of PC-based methods to modulate suppressive immunity would help to prevent immediate immune responses in stem cell-based therapy, allogeneic organs transplantation, and treatment of autoimmune and auto-inflammatory disorders.

## 5. Cancer Evokes Immunosuppressive Function in PC

The role of PCs in the tumour angiogenesis, metastasis, evasion of the host immune system, and resistance to anti-cancer therapy has attracted great interest [91,92]. Among many types of cancer, the effect of glioblastoma on PC is particularly important because glioblastoma-activated PCs develop an immunosuppressive phenotype, thus reducing T cell activation supporting glioblastoma growth in vitro and in vivo (reviewed in [93]). However, the role of PCs in tumour development and progression is complex. Despite contribution to immune evasion, they provide valuable targets for cancer immunotherapy. Further, we discuss recent results and implications of the PC-related findings in cancer biology.

Inoculation of breast tumour cells to the pdgfbret/ret PC-deficient mice model resulted in the development of defective tumour vasculature, followed by a more hypoxic microenvironment. Furthermore, hypoxia promoted IL-6 up-regulation in the tumour cells and increased transmigration of myeloid-derived suppressor cells (MDSCs), which are immature myeloid cells inhibiting T cell-mediated antitumour reactivity. Thus, MDSC accumulation in tumours promoted tumour growth, while PDGFB overexpression restored PC coverage and abolished the increased MDSC trafficking to PC-deficient tumours [94]. Another study demonstrated that PCs promoted metastasis through recruitment of tumour-associated macrophages (TAM) in an IL-33-dependent way. Therefore, IL-33 deletion blocked PDGF-B-induced TAM recruitment and metastasis [95].

**Figure 3:**
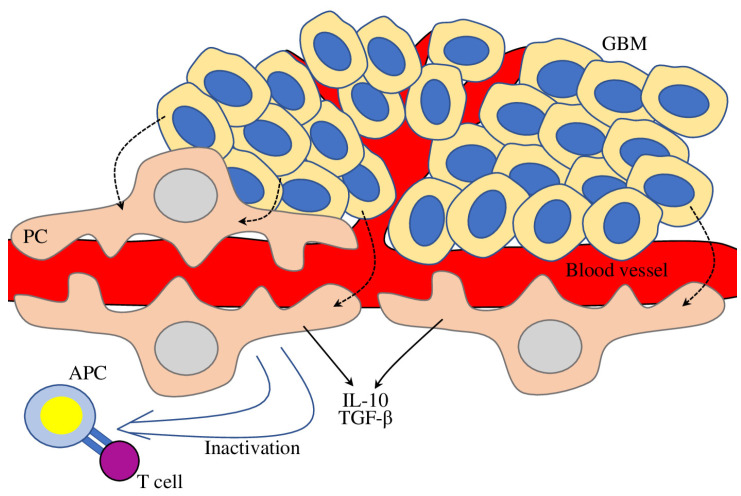
The role of pericytes in glioblastoma environment. GBM-affected PC (dashed arrows) increase production of anti-inflammatory molecules (IL-10, TGFβ, and MHC-II) (solid arrows), thus facilitation immunosuppression (hollow arrow) and tumour growth.

Glioblastoma multiforme (GBM) is a highly invasive cancer with several known immunosuppressive mechanisms, such as TAMs recruitment and modulation of CKs expression and anti-tumour T cell responses [96]. Interaction of brain PCs with GBM cells up-regulated the expression of anti-inflammatory cytokines TGFβ and IL-10, while the expression of co-stimulatory molecules (CD80 and CD86) and MHC-II molecules was significantly reduced, thus reducing T cell responses (Figure 3). Finally, in vivo experiments on mice with orthotopic xenotransplant of human GBM cells co-cultured with PC demonstrated increased perivascular infiltration of GBM cells and higher GBM tumour mass, confirming that GBC-PC immunosuppressive properties assisted GBM cell proliferation and tumour growth [97].

Recently, autophagy was proposed as another mechanism utilised by GBM cells through cell-co-cell interaction with PCs to modulate their immune function. Autophagy is the conserved process of degradation of the damaged and malfunctional cellular components in a lysosome-dependent way [98]. In particular, the activity of chaperone-mediated autophagy (CMA), which selectively degrades soluble cytosolic proteins containing the KFERQ motif in their sequence [99], was up-regulated in many cancer types, including GBM. Up-regulated CMA allows cancer cells to resist oxidative stress, degrade anti-oncogenes and negative regulators of cell proliferation, and suppress anti-tumour immune response [100]. CMA inhibition in cancer cells has anti-tumour activity: decreases cell proliferation rate and metastatic ability, and stimulates tumour antigen-specific T cell responses [98].

The incubation of PCs with GBM cells caused oxidative burst and CMA up-regulation in PCs, and maintained pro-inflammatory and immunosuppressive function. However, the co-incubation of GBM with PC with impaired CMA activity failed to suppress the ability of PC to activate T cell responses, to reduce the expression of the co-stimulatory molecule CD80 and to alter anti-inflammatory phenotype. Furthermore, in vivo transplantation of CMA-defective PCs into a GBM mice model demonstrated increased GBM cell death rate, reduced proliferation, and effective immune response compared with mice grafted with control PCs [101]. Transcriptomic profiling in CMA-deficient PCs in response to GBM cells co-incubation revealed the most affected pathways: up-regulated anti-tumour cell functions (cell-adhesion (CAMs), immune and inflammatory responses, phagosome formation), and down-regulated pro-tumoural functions (cell adhesion (adherent junctions), angiogenesis, regulation of actin cytoskeleton and others). Moreover, several bioactive molecules related to tumour immune responses were identified in the PC secretome dependent on GBM-induced CMA: periostin, lumican, vitamin D, gelsolin, and osteopontin. Finally, exofucosylated CMA-deficient PCs in the form of intracranial or intravenous therapy effectively reached the tumour site and activated the anti-tumour T cell responses [102].

In total, these findings identify that GBM interaction with PC is vital for tumour cells survival and progression. CMA is one of the key mechanisms, up-regulated by GBM in PC and necessary to elicit the immunosuppressive function of PC and further stabilise GBM–PC interactions. Moreover, several bioactive molecules related to the tumour immune responses were proposed as new markers, which can be used for GBM diagnostics and therapy. Finally, CMA ablation in PC was proposed as a basement for the development of future therapeutic approaches against GBM.

## 6. Conclusions

Recent studies have defined the critical roles of pericytes in regulating of innate and adaptive immunity. PCs respond to different pro-inflammatory stimuli by complex secretory responses, which promote the expression of a variety of pro-inflammatory and anti-inflammatory molecules. At the same time, PCs overexpress adhesion molecules that recruit and guide innate immune cells (such as macrophage, neutrophil, natural killer, and monocyte) to the inflammation sites after migration through vessel walls. Moreover, PCs implement different mechanisms to shape the adaptive immunity, among which immunosuppressive and immunomodulative roles are of particular interest to treat cancer and autoimmune disorders.

In recent years a very limited number of clinical trials have included pericyte-based research, such as lung diseases (chronic obstructive pulmonary disease (COPD)) [103,104], asthma [105], osteoarthritis [106], and cancer [107,108,109,110]. Unfortunately, none of them have directly investigated or applied pericyte-based immunomodulatory approaches. However, pericyte-based immunotherapy methods and techniques are superficially unexplored areas of research and further investigation is necessary to develop and implement them in the clinic.

In conclusion, PCs are important components of the immune response, which suggests that specifically targeting this cell type might be a promising approach for various diseases.

## Figures and Tables

**Table 1:** The common markers for pericytes identification.

Marker	Location	Function	Other Cell Types/Tissues/Organs	References
NG2	Arteriolar and capillary pericytes	Pericyte/endothelial cell interaction in tumour angiogenesis	Cancer cells, Oesophagus, Placenta, Uterus and others	[7]
*α*SMA	Capillary pericytes	Regulates contraction/relaxation	Smooth muscle cells	[8]
PDGFR-*β*	Brain pericytes	Pericytes recruitment during embryogenic angiogenesis	Fibroblasts and smooth muscle cells	[9]
RGS5	Brain pericytes in mouse embryogenic development	Tumour and embryogenic angiogenesis	Abundantly expressed in blood vessels, heart, lung, skeletal muscle, and small intestine	[10]
FOXD1^+^ - progeny	Lung and kidney pericytes	Contributes to the myofibroblast pool in kidney and pulmonary fibrosis	Lung and kidney perivascular cells and myofibroblasts	[11,12]

## Data Availability

Not applicable.

## Abbreviations

ACKR1	Atypical chemokine receptor 1
Ang-1/Tie-2	Angiopoietin 1/TEK Receptor Tyrosine Kinase
APC	Antigen-presenting cell
BBB	Blood–brain barrier
BDNF	Brain-derived neurotrophic factor
C/EBP	CCAAT enhancer binding protein
C1q	Complement C1q Chain A
C5aR1	Complement Component 5a Receptor 1
CD105	Endoglin
CD11b	Alpha chain of the integrin Mac-1/CR3
CD13	Aminopeptidase N
CD163	Macrophage-Associated Antigen
CD4	T Cell Surface Glycoprotein CD4
CD45	Leukocyte-common antigen
CD68	Macrophage Antigen CD68
CMA	Chaperone-mediated autophagy
COX2	Cyclooxygenase-2
DAMP	Damage-associated molecular patterns
EAE	Experimental auto-immune encephalomyelitis
ECs	Endothelial cells
FGFb	Basic fibroblast growth factor (b)
FOXP3	Forkhead box P3
FSP	Fibroblast Secretory Protein
G-CSF	Granulocyte colony-stimulating factor
GM-CSF	Granulocyte-macrophage colony-stimulating Factor
GROa/b/g	Growth-regulated protein alpha/beta/gamma
HMGB1	High mobility group box 1
HPSCs	Human pluripotent stem cells
I/R	Ischaemia-reperfusion
ICAM-1	Intercellular Adhesion Molecule 1
IDO1	Indoleamine 2,3-dioxygenase 1
IFN-γ	Interferon Gamma
Jag1	Jagged Canonical Notch Ligand 1
LCFAs	Free long-chain fatty acids
LPS	Lipopolysaccharide
MHC	Major histocompatibility complex
MCP-1	Monocyte Chemoattractant Protein-1
MCSF	Macrophage colony-stimulating factor
MDSC	Myeloid-derived suppressor cells
MIF	Macrophage migration–inhibitory factor
MIP-1α	Macrophage Inflammatory Protein-1-Alpha
MMP2	Matrix Metallopeptidase 2
MSCs	Multipotent mesenchymal stem cells
NG2	Nerveglial antigen-2/chondroitin sulfate proteoglycan 4
NLRs	NOD-like receptors
NOD1	Nucleotide Binding Oligomerization Domain Containing 1
Notch3	Notch Receptor 3
NOX4	NADPH Oxidase 4
PC	Pericytes
PDGF-BB	Platelet-derived growth factor-BB
PDGFR-β	Platelet-derived growth factor receptor β
PD-L1	Programmed Cell Death 1 Ligand 1
PGN	Peptidoglycan
PLGF	Placental growth factor
PRR	Pattern-recognition receptors
RGS5	The regulator of G-protein signaling-5
RIPK2	Receptor Interacting Serine/Threonine Kinase 2
SDF-1a	Stromal cell-derived factor 1 alpha
SMAD2/3	Mad-Related Protein
SMCs	Smooth muscle cells
SOD2	Superoxide Dismutase 2
TAM	Tumour-associated macrophages
TEM	Effector memory T cells
TGFβ1	Transforming Growth Factor Beta 1
TJ	Tight junction
TLRs	Toll-like receptors
TNF-α	Tumour Necrosis Factor-Alpha
Tregs	Regulatory T cells
uNK	Uterine natural killer
VAP-1	Vascular Adhesion Protein-1
VCAM1	Vascular Cell Adhesion Protein-1
VEGF	Vascular endothelial growth factor
αSMA	α-smooth muscle actin
βNGF	Nerve growth factor beta
